# Astaxanthin Supplementation Improves the Subsequent Developmental Competence of Vitrified Porcine Zygotes

**DOI:** 10.3389/fvets.2022.871289

**Published:** 2022-04-01

**Authors:** Decai Xiang, Baoyu Jia, Bin Zhang, Jiachong Liang, Qionghua Hong, Hongjiang Wei, Guoquan Wu

**Affiliations:** ^1^National Regional Genebank (Yunnan) of Livestock and Poultry Genetic Resources, Yunnan Provincial Engineering Laboratory of Animal Genetic Resource Conservation and Germplasm Enhancement, Yunnan Animal Science and Veterinary Institute, Kunming, China; ^2^Key Laboratory for Porcine Gene Editing and Xenotransplantation in Yunnan Province, College of Veterinary Medicine, Yunnan Agricultural University, Kunming, China

**Keywords:** astaxanthin, pig, zygote, vitrification, embryonic development

## Abstract

Cryopreservation of embryos has been confirmed to cause oxidative stress as a factor responsible for impaired developmental competence. Currently, astaxanthin (Ax) raises considerable interest as a strong exogenous antioxidant and for its potential in reproductive biology. The present study aimed to investigate the beneficial effects of Ax supplementation during *in vitro* culture of vitrified porcine zygotes and the possible underlying mechanisms. First, the parthenogenetic zygotes were submitted to vitrification and then cultured in the medium added with various concentrations of Ax (0, 0.5, 1.5, and 2.5 μM). Supplementation of 1.5 μM Ax achieved the highest blastocyst yield and was considered as the optimal concentration. This concentration also improved the blastocyst formation rate of vitrified cloned zygotes. Moreover, the vitrified parthenogenetic zygotes cultured with Ax exhibited significantly increased mRNA expression of *CDX2, SOD2*, and *GPX4* in their blastocysts. We further analyzed oxidative stress, mitochondrial and lysosomal function in the 4-cell embryos and blastocysts derived from parthenogenetic zygotes. For the 4-cell embryos, vitrification disturbed the levels of reactive oxygen species (ROS) and glutathione (GSH), and the activities of mitochondria, lysosome and cathepsin B, and Ax supplementation could fully or partially rescue these values. The blastocysts obtained from vitrified zygotes showed significantly reduced ATP content and elevated cathepsin B activity, which also was recovered by Ax supplementation. There were no significant differences in other parameters mentioned above for the resultant blastocysts. Furthermore, the addition of Ax significantly enhanced mitochondrial activity and reduced lysosomal activity in resultant blastocysts. In conclusion, these findings revealed that Ax supplementation during the culture period improved subsequent embryonic development and quality of porcine zygotes after vitrification and might be used to ameliorate the recovery culture condition for vitrified embryos.

## Introduction

Cryopreservation of gametes and embryos is an important technique for the long-term conservation and dissemination of genetic resources as well as animal breeding and human-assisted reproductive technologies (1, 2). Currently, vitrification is regarded as a most common and effective way to cryopreserve porcine embryos in different developmental stages (3, 4). It is well known that vitrification of embryos at the zygote stage is a convenient strategy for their effective utilization, such as implementing embryo transfer and obtaining embryonic stem cells without any time and space restrictions. In our previous studies, although porcine zygotes derived from parthenogenetic activation (PA) and somatic cell nuclear transfer (SCNT) have been successfully vitrified with high cryosurvival, their developmental competence has not yet been satisfactory (5, 6). Some sublethal damages induced by vitrification are likely to remain in the subsequent embryo development of porcine zygotes. So, it is necessary to reduce or eliminate these cryodamages during *in vitro* culture (IVC) of the vitrified zygotes, in order to improve yield and quality of blastocysts. A vast of studies have reported that cryopreservation causes an increase in oxidative stress by overproduction of reactive oxygen species (ROS) in oocytes and embryos, which may be responsible for their impaired quality and development (7). Because the excessive amounts of ROS eventually result in changes at the physiological, biochemical and molecular levels, including DNA fragmentation, spindle defect, metabolism abnormality, organelle dysfunction, apoptosis and so on (8, 9). Furthermore, various antioxidants can be more effective in inhibiting oxidative stress of oocytes and embryos during cryopreservation and/or subsequent culture *in vitro*. Commonly used antioxidants such as melatonin (10, 11), resveratrol (12, 13), glutathione (14, 15) have been shown to enhance the oocyte and embryo potential after cryopreservation.

Astaxanthin (Ax) is an oxidized derivative of carotenoids that exists everywhere in nature including several marine animals (e.g., crab, salmon, and shrimp), plants, microbes, and microalgae (16). Its antioxidant activity has been proved to be much higher than that of α-tocopherol, β-carotene, lutein, vitamin C, and lycopene (17, 18). Based on the excellent antioxidant property, Ax has been recognized as a potential nutraceutical owing to its anti-cancer, anti-diabetic, anti-inflammatory and anti-obesity (19, 20). Several studies have demonstrated the beneficial effects of Ax in the reproductive biology of mammalian species. For instance, Ax supplementation can improve the bovine oocyte maturation and embryo development during *in vitro* maturation (IVM) and IVC (21), and enhance the quality of bovine oocytes during *in vitro* growth (22). Moreover, treatment with Ax is found to protect against the developmental impairment of oocytes and embryos induced by deleterious factors including heat stress and oxidative stress (23–26). Our previous studies have also pointed out that Ax improves the maturation quality of porcine immature oocytes after vitrification, and inhibits the porcine oocyte aging *in vitro* (27, 28). However, whether Ax may protect the vitrified zygotes through its antioxidant capacity remains unclear. Therefore, the aim of the present study was to confirm the cytoprotective effects of Ax supplementation during the IVC process on vitrified porcine zygotes and explore the possible mechanisms.

## Materials and Methods

All chemicals and reagents used in this study were purchased from Sigma-Aldrich Chemical Company (Shanghai, China), except for those specifically mentioned. Tissue culture medium-199 (TCM-199), Dulbecco's phosphate buffered saline (DPBS), Dulbecco's modified Eagle's medium (DMEM), knockout serum replacement, CM-H_2_DCFDA, ThiolTracker™ Violet, MitoTracker™ Red CMXRos, BODIPY FL ATP, LysoTracker™ Red were obtained from ThermoFisher Scientific (Shanghai, China).

### Oocyte Collection and Maturation *in vitro*

Porcine ovaries from prepubertal gilts were obtained at a local slaughterhouse, stored in physiological saline maintained at 35–37°C, and transported to the laboratory within 2 h. Follicular fluids were aspirated from 3–8 mm antral follicles using a 20-mL disposable syringe with an 18-gauge needle, and then injected into a 15 mL conical tube for precipitating the cumulus-oocyte complexes (COCs). Thereafter, the sediments were washed two times with Tyrode's lactate-HEPES-polyvinyl alcohol (TLH-PVA) medium (29). The COCs were picked up under a stereomicroscope (Olympus, Tokyo, Japan). Only COCs with uniform cytoplasm and surrounding cumulus cells were selected for IVM. After washing three times with IVM medium, ~50–70 COCs were transferred to 500 μL of the medium in each well of a 24-well plate (Costar, Corning, NY) and then cultured for 42–44 h at 39°C in a humidified atmosphere of 5% CO_2_. The IVM medium was TCM-199 supplemented with 10% (v/v) porcine follicular fluid, 3.05 mM D-glucose, 0.57 mM cysteine, 0.91 mM sodium pyruvate, 10 ng/mL epidermal growth factor, 0.5 μg/mL each follicle-stimulating hormone and luteinizing hormone. After IVM, cumulus cells of COCs were removed by repeated pipetting in TLH-PVA medium containing 0.1% (w/v) hyaluronidase. The oocytes were selected for the following experiments only if they had a first polar body and evenly dark cytoplasm.

### PA, SCNT, and Embryo Culture *in vitro*

All procedures for PA and SCNT were performed as previously reported (30). For PA, oocytes were equilibrated for about 15 s in activation medium (0.28 M mannitol, 0.1 mM MgSO_4_, 0.05 mM CaCl_2_, and 0.5 mM HEPES), then placed between two wires of a microslide 0.5 mm fusion chamber. Subsequently, they were stimulated with a direct current (DC) pulse of 130 V/mm for 80 μs using a BLS CF-150/B cell fusion machine (BLS, Budapest, Hungary), and cultured in porcine zygote medium-3 (PZM-3) (31) supplemented with 5 μg/mL cytochalasin B for 4 h at 39°C in a humidified atmosphere of 5% CO_2_.

For SCNT, the ear tissues were obtained from Diannan miniature pigs. Cells from a single batch were cultured in DMEM supplemented with 10% fetal bovine serum (Ausbian, Sydney, Australia) at 38.5°C in a humidified atmosphere of 5% CO_2_ until the cells formed a monolayer. Then, donor cells were harvested with trypsin for SCNT when they were synchronized at the G0/G1 phase after four to eight passages.

Furthermore, oocytes were incubated in PZM-3 containing 0.1 μg/mL demecolcine and 0.05 M sucrose for 0.5–1 h. Next, the oocytes were enucleated by gentle aspirating the first polar body and chromosomes using a beveled pipette, and a donor cell was inserted into the perivitelline space. The reconstructed cell-oocyte couplets were placed in fusion medium (0.28 M mannitol, 0.1 mM MgSO_4_, and 0.5 mM HEPES) for fusing by a DC pulse of 200 V/mm for 20 μs using an Electro Cell Fusion Generator (LF201, NEPA GENE Co., Ltd., Japan). Subsequently, they were incubating in PZM-3 for 0.5–1 h and activated in activation medium with a DC pulse of 150 V/mm for 100 μs, and then cultured in PZM-3 containing 5 μg/mL cytochalasin B for 2–4 h at 39°C in a humidified atmosphere of 5% CO_2_.

After washing in PZM-3, the presumptive PA and SCNT embryos were cultured in this medium under conditions described above. The cleavage and blastocyst formation rates were examined on 48 and 144 h, respectively. To determine total cell member, the resultant blastocysts were stained for10 min with 10 μg/mL Hoechst 33342 in DPBS containing 0.3% (w/v) polyvinyl alcohol (DPBS-PVA), and observed using an inverted fluorescence microscope (Nikon, Tokyo, Japan) with ultraviolet light.

### Vitrification and Warming

After chemical activation, the presumptive zygotes were used to vitrification and warming according to a previous report (6), in a laboratory maintained at 25 ± 1°C. Basic medium (BM) was prepared using DPBS supplemented with 20% (v/v) knockout serum replacement, which was used to make up the equilibration solution, vitrification solution and warming solution. After washing in BM for 3 min, the zygotes were first equilibrated with equilibration solution containing 5% (v/v) ethylene glycol (EG) for 3 min, then transferred into vitrification solution [0.6 M sucrose, 50 mg/mL polyvinylpyrrolidone and 35% (v/v) EG] for 20–30 s at 25°C. Approximately10 zygotes each group were loaded onto tip of a Cryotop carrier (Kitazato Biopharma, Shizuoka, Japan) with the minimum volume of vitrification solution. The Cryotop was quickly plunged into liquid nitrogen (LN_2_) and covered with a plastic cap. For warming, the zygotes located on Cryotop were rapidly transferred from LN_2_ into 42°C warming solution containing 1.0 M sucrose, and kept for 1 min on a 42°C hot plate. Next, they were transferred stepwise into dilution warming solution containing 0.5 and 0.25 M sucrose at 39°C for 2.5 min, respectively. After keeping in BM for 5 min, these vitrified zygotes were transferred to PZM-3 with or without Ax, and continued to complete 144 h of embryo culture. After 2 h of warming, the survival rate of vitrified zygotes was assessed under a stereomicroscope based on their morphological alterations.

### Fluorescent Staining

After two washes in DPBS-PVA, the 4-cell embryos and blastocysts were incubated for 30 min at 39°C with 10 μM CM-H_2_DCFDA for ROS imaging, with 10 μM ThiolTracker™ Violet for glutathione (GSH) imaging, with 200 nM MitoTracker™ Red CMXRos for mitochondria imaging, with 50 nM for lysosome imaging, or with 1:250 MR-RR2 (Enzo Life Sciences, Farmingdale, NY, USA) for cathepsin B imaging. Subsequently, the stained oocytes were washed three times in DPBS-PVA and then imaged under a confocal laser-scanning microscope (Nikon A1, Tokyo, Japan). To assess the ATP content, embryos were fixed with 4% paraformaldehyde for 2 h and then incubated in DPBS-PVA supplemented with 500 nM BODIPY FL ATP for 1 h at room temperature. The detection method was the same as those described above. For each fluorescent probe, the fluorescence intensity was measured with the same scan settings and analyzed using NIS-Elements software (Nikon, Tokyo, Japan). Fluorescence intensity in the fresh control group was set arbitrarily at 1, and fluorescence intensity in each treatment group was expressed as relative values to the fresh control group.

### Quantitative Real-Time PCR (qRT-PCR)

Total RNA was reverse transcribed into cDNA from the blastocysts (5 per group) using a TransScript®-Uni Cell to cDNA Synthesis SuperMix for qPCR (TransGen Biotech, Beijing, China) according to the manufacturer's protocol. qRT-PCR reactions were conducted using a CFX Real-Time PCR Detection System (Bio-Rad, Hercules, CA, USA) in a 20 μL mixture containing 2 μL cDNA, 0.8 μL forward and reverse primers, 10 μL Fast qPCR Mix SYBR Green I (Tsingke, Beijing, China) and nuclease-free water. Reaction condition consisted of the following: 95°C for 1 min, followed by 40 cycles of 95°C for 10 s and 60°C for 15 s. Each group had four biological replicates and each reaction was replicated three times. Relative gene expression levels were quantified by the 2^−ΔΔCT^ method using GAPDH mRNA as normalization. Primer sequences used are listed in Supplementary Table S1.

### Statistical Analysis

Percentage data were arcsine transformed before analysis to ensure homogeneity of variance. All data were analyzed using SPSS 20.0 software (SPSS Inc., Chicago, IL, USA) with one-way ANOVA followed by Student–Newman–Keulsa's multiple comparison test. Results were presented as the least-squares mean ± SEM, and *P* < 0.05 indicated a significant difference.

## Results

### Effects of Ax Supplementation on Developmental Competence

Firstly, to evaluate the potential effects of Ax supplementation on developmental competence of vitrified zygotes following PA, they were cultured in the PZM-3 medium supplemented with various concentrations of Ax (0, 0.5, 1.5, and 2.5 μM). After 2 h of warming, the survival rate in all treatment groups was similar to that of the fresh control group (data not shown). As shown in Table 1, the cleavage rate was similar (*P* > 0.05) among all groups. Moreover, the blastocyst formation rate in the 1.5 μM Ax group was significantly higher (*P* < 0.05) than in the vitrified control group, but still was significantly lower (*P* < 0.05) than in the fresh control group. There was no significant difference (*P* > 0.05) in the total cell number of blastocyst in all groups.

**Table 1:** Effects of astaxanthin (Ax) supplementation (0.5, 1.5, and 2.5 μm) on developmental competence of vitrified zygotes derived from parthenogenetic activation.

**Groups**	**No. zygotes cultured**	**Cleavage rate (%)**	**Blastocyst rate (%)**	**No. cells per blastocyst**
Fresh	197	93.4 ± 1.0	62.5 ± 3.7^a^	50.7 ± 3.1
Vitr	206	87.5 ± 1.6	41.5 ± 2.8^c^	46.8 ± 4.2
Vitr + 0.5 μm Ax	208	88.8 ± 2.1	42.5 ± 1.9^c^	48.1 ± 3.3
Vitr + 1.5 μm Ax	215	88.5 ± 1.4	51.7 ± 1.6^b^	48.9 ± 3.7
Vitr + 2.5 μm Ax	211	89.6 ± 1.0	45.5 ± 1.8^b^^c^	45.6 ± 4.0

*Four replications were performed*.

*Data are expressed as the mean ± SEM values*.

a−c *Within a column, means without a common superscript differed (P < 0.05)*.

Based on the above results, the optimal concentration of Ax was determined to be 1.5 μM. In the following experiments, the zygotes were randomly divided into three experimental groups: fresh zygotes (Fresh group), vitrified zygotes (Vitr group), and vitrified zygotes cultured with 1.5 μM Ax (Vitr + Ax group). Furthermore, we also examined whether Ax supplementation could improve the developmental competence of vitrified zygotes following SCNT. Similarly, the Vitr + Ax group showed significantly increased (*P* < 0.05) blastocyst formation rate compared with the Vitr group, and the value also was significantly lower (*P* < 0.05) than that of the Fresh group (Table 2).

**Table 2:** Effects of astaxanthin (Ax) supplementation (1.5 μm) on developmental competence of vitrified zygotes derived from somatic cell nuclear transfer.

**Groups**	**No. zygotes cultured**	**Cleavage rate (%)**	**Blastocyst rate (%)**	**No. cells per blastocyst**
Fresh	108	73.1 ± 2.3	28.7 ± 1.2^a^	41.3 ± 4.3
Vitr	101	71.3 ± 2.4	17.8 ± 1.6^c^	38.9 ± 4.8
Vitr + 1.5 μm Ax	103	71.9 ± 2.1	23.3 ± 1.3^b^	40.4 ± 4.4

*Three replications were performed*.

*Data are expressed as the mean ± SEM values*.

a−c *Within a column, means without a common superscript differed (P < 0.05)*.

### Effect of Ax Supplementation on mRNA Expression Levels in Resultant Blastocysts

**Figure 1:**
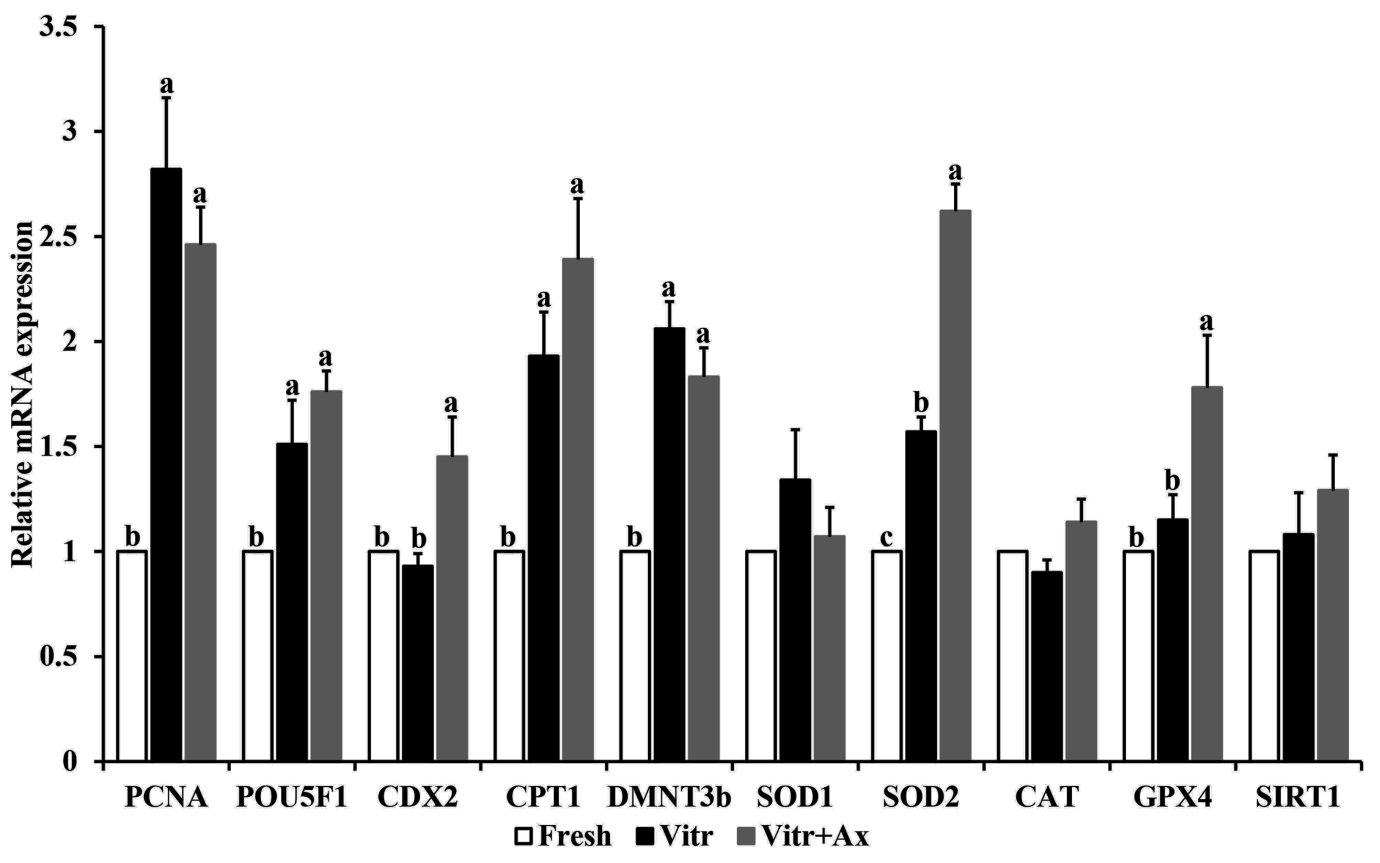
Effects of astaxanthin (Ax) supplementation on mRNA expression levels of genes related to embryo development and oxidative stress in resultant blastocysts. Four replications were performed. Data are the mean ± SEM values. Different superscripts above columns indicate significant differences (*P* < 0.05). The three experimental groups: fresh zygotes (Fresh group), vitrified zygotes (Vitr group), and vitrified zygotes cultured with Ax (Vitr + Ax group).

To further confirm the effects of Ax supplementation on the quality of resultant blastocysts, qRT-PCR was submitted to detect the gene expression related to embryonic development and antioxidant defense (*PCNA, POU5F1, CDX2, CPT1, DMNT3b, SOD1, SOD2, CAT, GPX4*, and *SIRT1*) in parthenogenetic blastocysts (Figure 1). There were no significant differences (*P* > 0.05) in mRNA expression of *SOD1, CAT* and *SIRT1* among all groups. The mRNA levels of *PCNA, POU5F1*, CPT1, and *DMNT3b* were similar (*P* > 0.05) between the Vitr and Vitr + Ax groups, and these values were significantly higher (*P* < 0.05) than those in the Fresh group. Moreover, the Vitr + Ax group had significantly increased (*P* < 0.05) mRNA levels of *CDX2, SOD2*, and *GPX4* as compared to the Fresh and Vitr groups, and the *SOD2* gene showed a significant higher (*P* < 0.05) expression in the Vitr group than in the Fresh group.

### Effects of Ax Supplementation on Oxidative Stress in Resultant 4-Cell Embryos and Blastocysts

**Figure 2:**
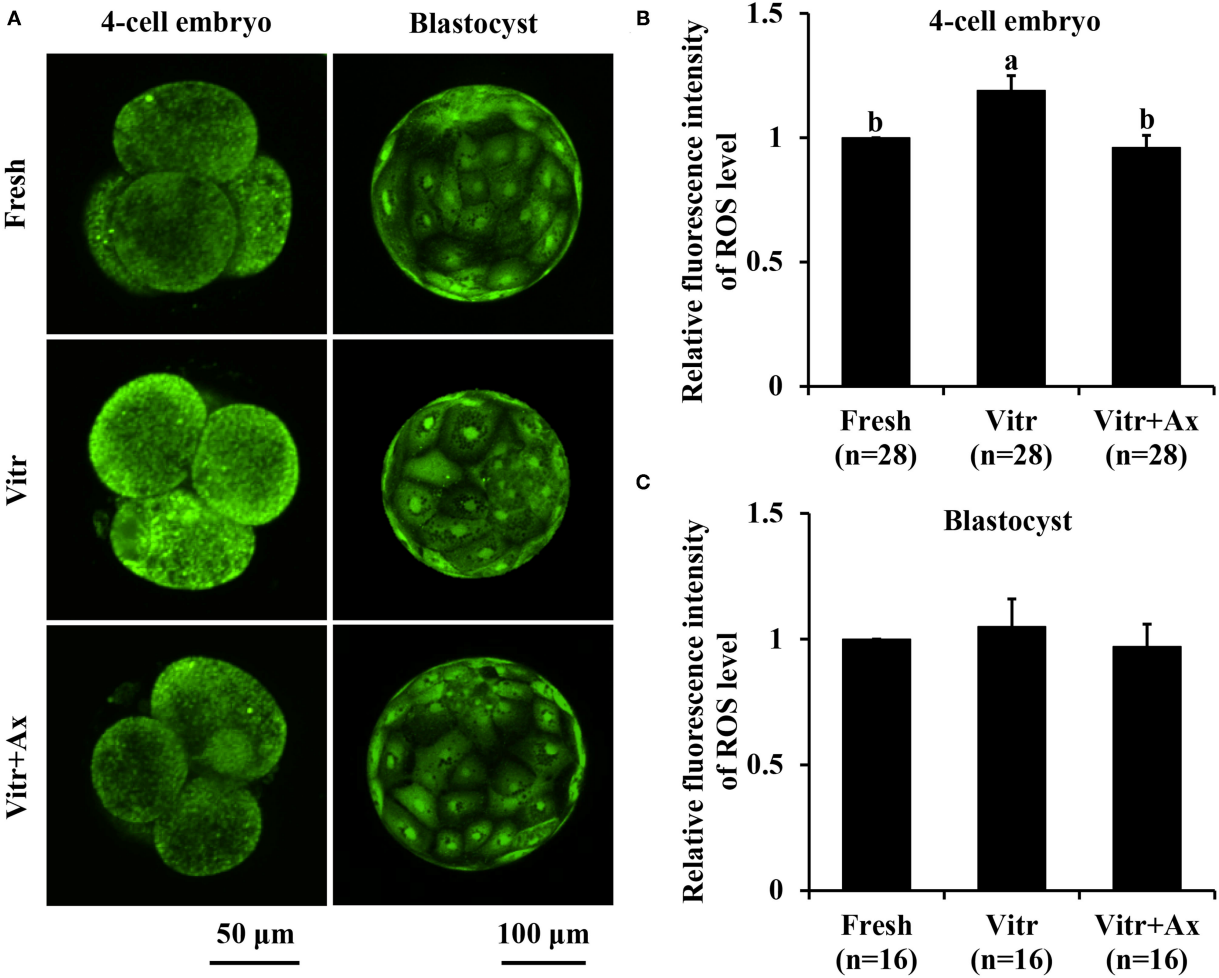
Effects of astaxanthin (Ax) supplementation on intracellular reactive oxygen species (ROS) level in resultant 4-cell embryos and blastocysts. **(A)** Representative images of ROS signals stained with CM-H_2_DCFDA. **(B,C)** Graphical representation of ROS level by quantifying the relative fluorescence intensity. Four replications were performed. Data are the mean ± SEM values. Different superscripts above columns indicate significant differences (*P* < 0.05). The three experimental groups: fresh zygotes (Fresh group), vitrified zygotes (Vitr group), and vitrified zygotes cultured with Ax (Vitr + Ax group).

**Figure 3:**
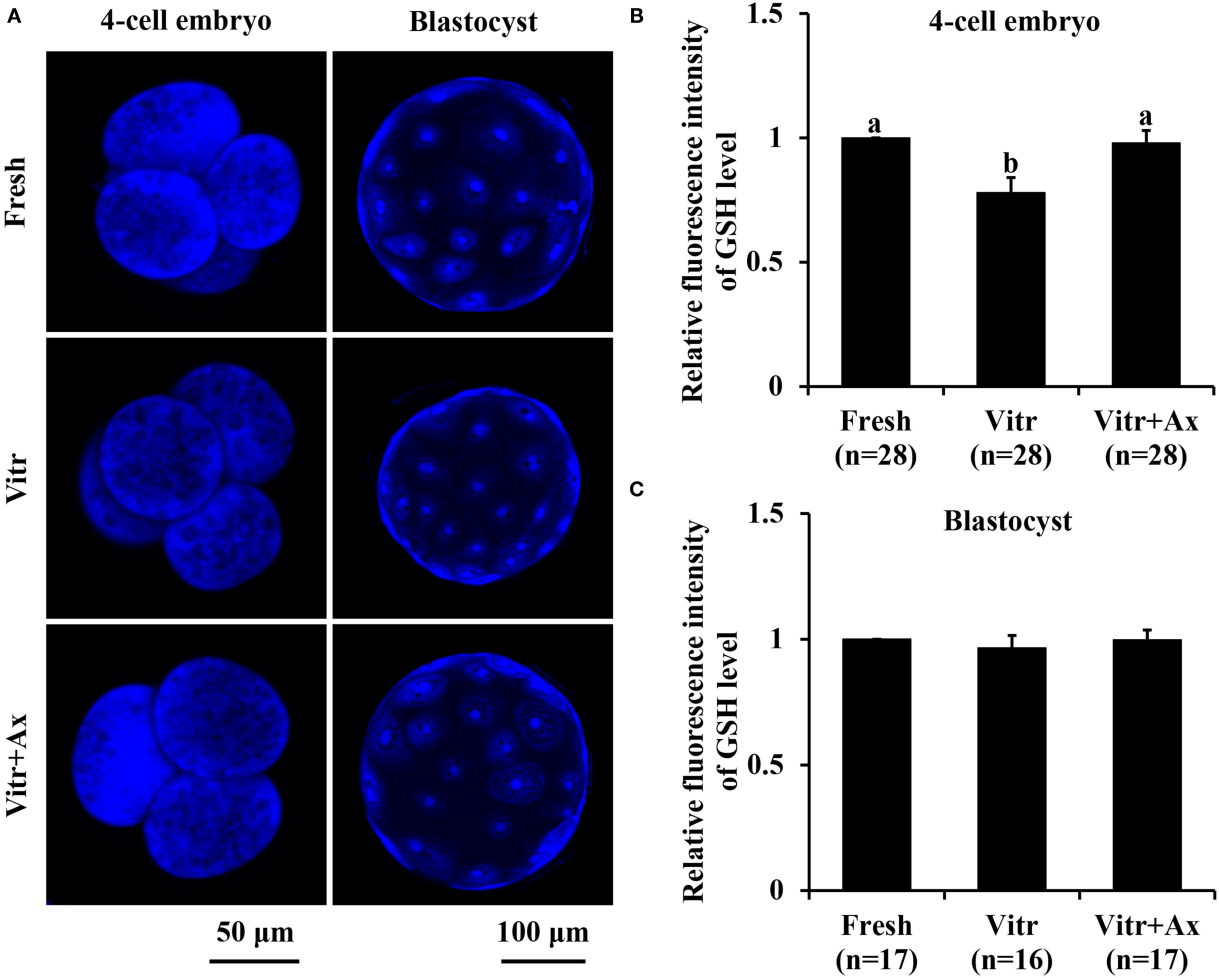
Effects of astaxanthin (Ax) supplementation on intracellular glutathione (GSH) level in resultant 4-cell embryos and blastocysts. **(A)** Representative images of GSH signals stained with ThiolTracker™ Violet. **(B,C)** Graphical representation of GSH level by quantifying the relative fluorescence intensity. Four replications were performed. Data are the mean ± SEM values. Different superscripts above columns indicate significant differences (*P* < 0.05). The three experimental groups: fresh zygotes (Fresh group), vitrified zygotes (Vitr group), and vitrified zygotes cultured with Ax (Vitr + Ax group).

To better understand the underlying mechanism of Ax supplementation during IVC, we analyzed oxidative stress, mitochondrial and lysosomal function in the 4-cell embryos and blastocysts, and parthenogenetic zygotes were used in the following experiments. Firstly, the levels of intracellular ROS and GSH were detected to reflect the degree of cellular oxidative stress. For the 4-cell embryos, there was no significant difference (*P* > 0.05) in ROS level between the Fresh and Vitr+Ax groups, and these values were significantly lower (*P* < 0.05) than that of the Vitr group (Figures 2A,B). Moreover, the ROS level of blastocysts was not different in all groups (Figures 2A,C). The GSH level of 4-cell embryos in the Vitr + Ax group was similar (*P* > 0.05) to that in the Fresh group, and was significantly higher (*P* < 0.05) than that in the Vitr group (Figures 3A,B). There was no significant difference (*P* > 0.05) in GSH level of blastocysts among all groups (Figures 3A,C).

### Effects of Ax Supplementation on Mitochondrial Function in Resultant 4-Cell Embryos and Blastocysts

**Figure 4:**
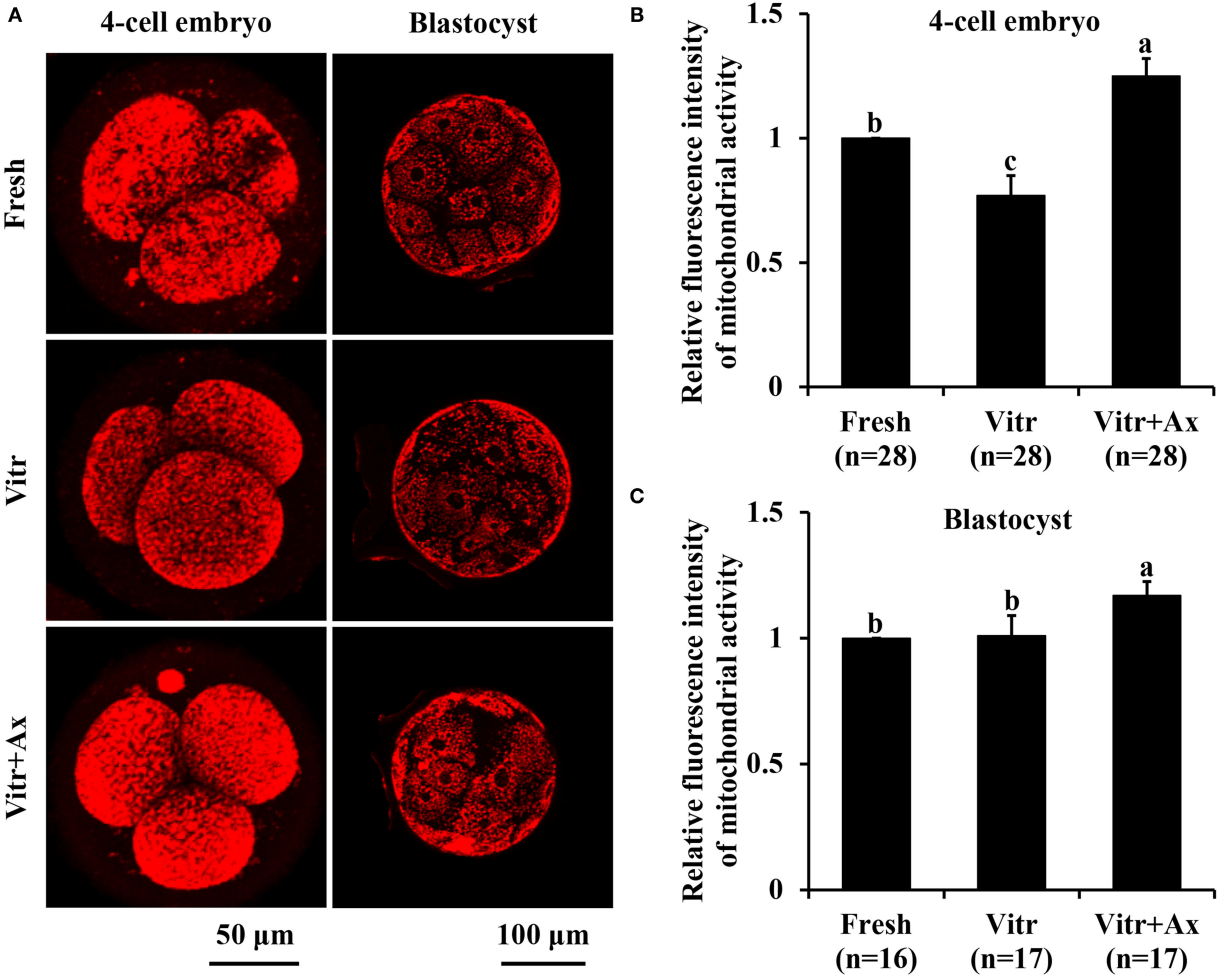
Effects of astaxanthin (Ax) supplementation on intracellular mitochondrial activity in resultant 4-cell embryos and blastocysts. **(A)** Representative images of mitochondrial signals stained with MitoTracker™ Red CMXRos. **(B,C)** Graphical representation of mitochondrial activity by quantifying the relative fluorescence intensity. Four replications were performed. Data are the mean ± SEM values. Different superscripts above columns indicate significant differences (*P* < 0.05). The three experimental groups: fresh zygotes (Fresh group), vitrified zygotes (Vitr group), and vitrified zygotes cultured with Ax (Vitr + Ax group).

**Figure 5:**
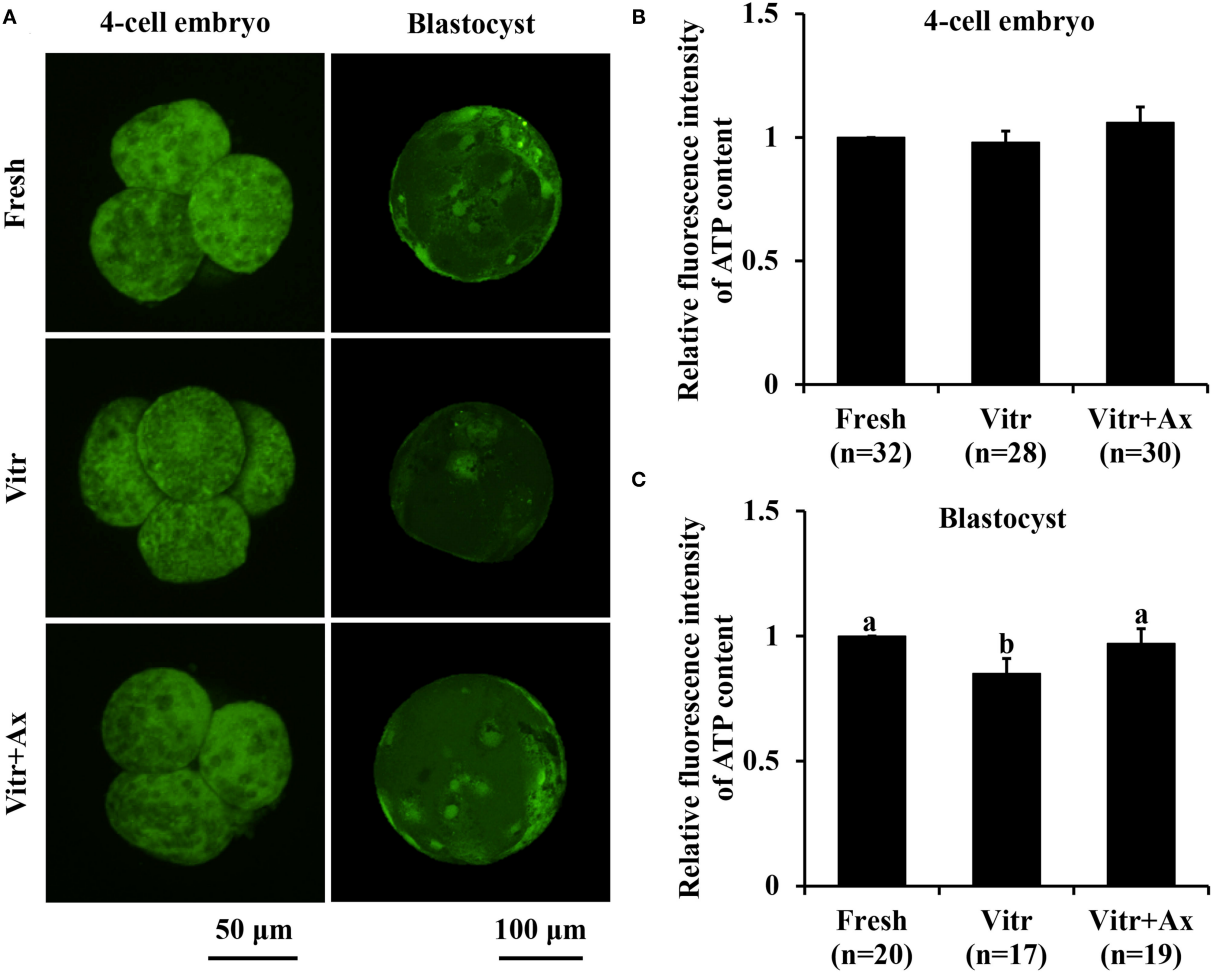
Effects of astaxanthin (Ax) supplementation on intracellular ATP content in resultant 4-cell embryos and blastocysts. **(A)** Representative images of ATP signals stained with BODIPY FL ATP. **(B,C)** Graphical representation of ATP content by quantifying the relative fluorescence intensity. Four replications were performed. Data are the mean ± SEM values. Different superscripts above columns indicate significant differences (*P* < 0.05). The three experimental groups: fresh zygotes (Fresh group), vitrified zygotes (Vitr group), and vitrified zygotes cultured with Ax (Vitr + Ax group).

Next, mitochondrial function in the 4-cell embryos and blastocysts was measured according to mitochondrial activity and ATP content. The mitochondrial activity of 4-cell embryos in the Vitr group was significantly reduced (*P* < 0.05) than that in the Fresh group, and the value was the highest (*P* < 0.05) in the Vitr + Ax group (Figures 4A,B). For the blastocysts, mitochondrial activity did not differ between the Fresh and Vitr groups, was significantly lower (*P* < 0.05) as compared to the Vitr + Ax group (Figures 4A,C). On the other hand, the ATP content of 4-cell embryos was similar (*P* > 0.05) among all groups (Figures 5A,B). However, the blastocysts in the Vitr group showed a significant decrease (*P* < 0.05) in ATP content when compared with the Fresh and Vitr + Ax groups (Figures 5A,C).

### Effects of Ax Supplementation on Lysosomal Function in Resultant 4-Cell Embryos and Blastocysts

**Figure 6:**
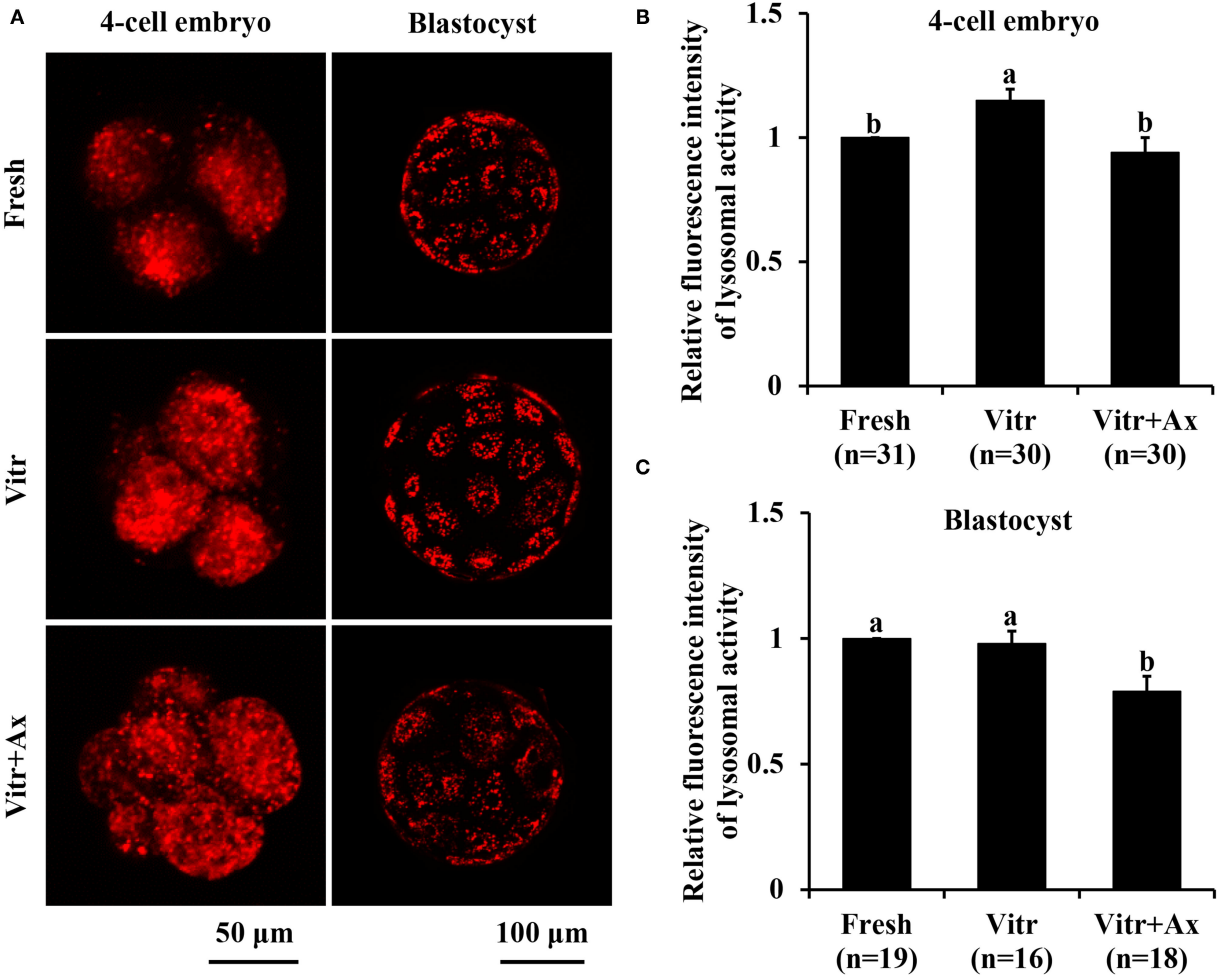
Effects of astaxanthin (Ax) supplementation on intracellular lysosomal activity in resultant 4-cell embryos and blastocysts. **(A)** Representative images of lysosomal signals stained with LysoTracker™ Red. **(B,C)** Graphical representation of lysosomal activity by quantifying the relative fluorescence intensity. Four replications were performed. Data are the mean ± SEM values. Different superscripts above columns indicate significant differences (*P* < 0.05). The three experimental groups: fresh zygotes (Fresh group), vitrified zygotes (Vitr group), and vitrified zygotes cultured with Ax (Vitr + Ax group).

**Figure 7:**
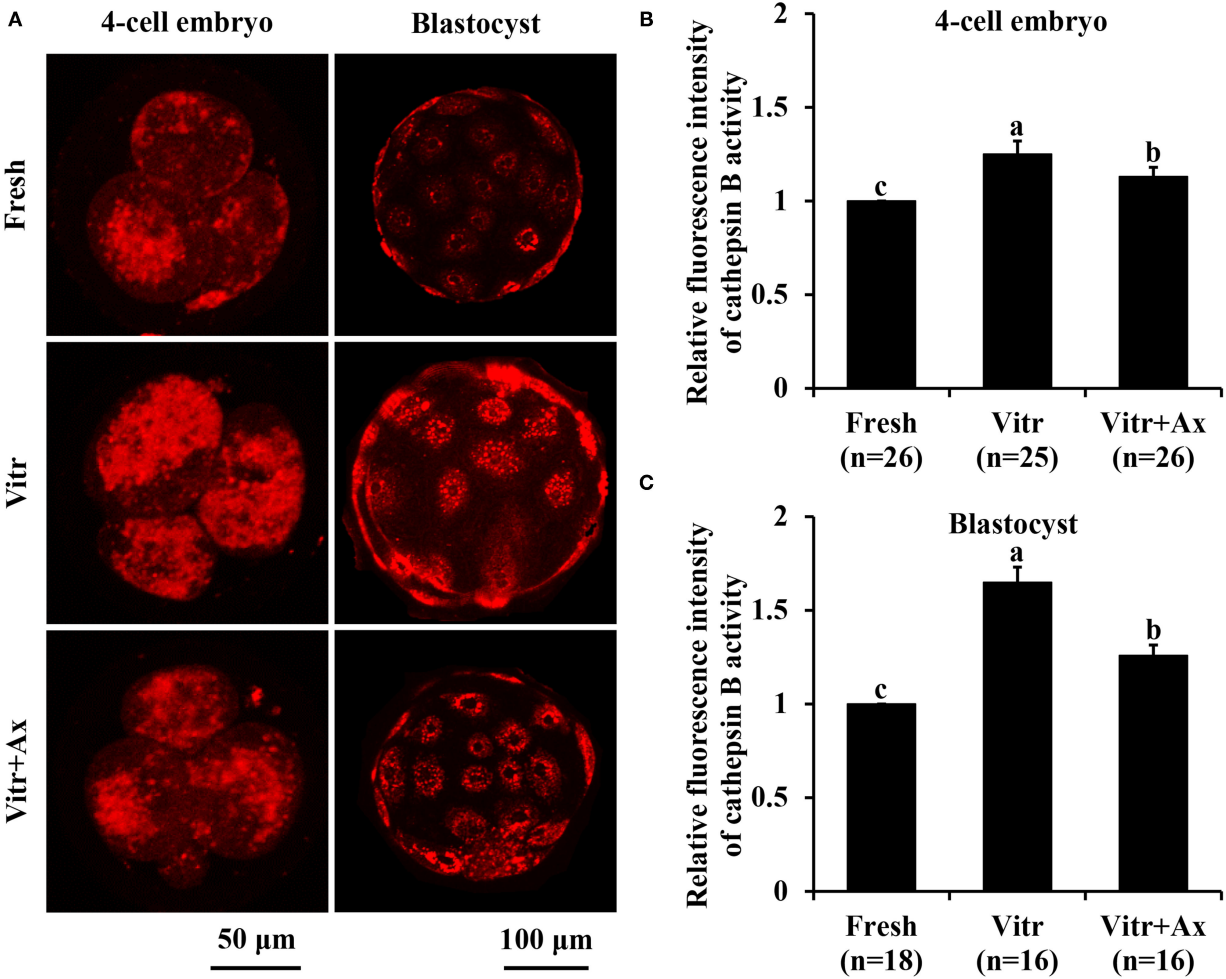
Effects of astaxanthin (Ax) supplementation on intracellular cathepsin B activity in resultant 4-cell embryos and blastocysts. **(A)** Representative images of cathepsin B signals stained with MR-RR2. **(B,C)** Graphical representation of cathepsin B activity by quantifying the relative fluorescence intensity. Four replications were performed. Data are the mean ± SEM values. Different superscripts above columns indicate significant differences (*P* < 0.05). The three experimental groups: fresh zygotes (Fresh group), vitrified zygotes (Vitr group), and vitrified zygotes cultured with Ax (Vitr + Ax group).

We finally investigated the lysosomal function in the 4-cell embryos and blastocysts by detecting the activities of lysosomes and cathepsin B. The lysosomal activity of 4-cell embryos was significantly increased (*P* < 0.05) in the Vitr group than in the Fresh and Vitr + Ax groups (Figures 6A,B). Moreover, there was similar (*P* > 0.05) in lysosomal activity of blastocysts between the Fresh and Vitr groups, and they were significantly higher (*P* < 0.05) as compared to the Vitr + Ax group (Figures 6A,C). For both 4-cell embryos and blastocysts, the cathepsin B activity in the Vitr + Ax group was significantly lower (*P* < 0.05) than in the Vitr group, but still significantly higher (*P* < 0.05) than in the Fresh group (Figures 7A–C).

## Discussion

Currently, porcine early-stage embryos after vitrification have compromised ability to develop *in vitro* to the blastocyst stage. One approach to improve blastocyst yield and quality can be to alleviate cellular and molecular damages induced by vitrification through optimize the culture condition. For example, the developmental competence of post-warming embryos has been confirmed to be enhanced by supplementation of the culture medium with exogenous reagents such as resveratrol (32, 33) or ZVAD-FMK (34). In our previous study, we have investigated the beneficial role of Ax treatment on maturation and development of porcine immature oocytes during vitrification and IVM processes (28). The present study also found that Ax supplementation during IVC exhibited cytoprotective effects against cryodamages in the vitrified porcine zygotes.

It has been reported that 0.5 mg/L Ax significantly improved development and epigenetic modifications for bovine cloned embryos (21). Treatment with Ax at the concentration of 500 μM enhances developmental ability of bovine IVF embryos co-cultured with bovine oviduct epithelial cell (23). In order to obtain the best result, an optimal concentration of Ax is needed. Our study showed that among assessed concentrations, 1.5 μM Ax supplementation during IVC achieved the highest blastocyst yield for porcine parthenogenetic zygotes after vitrification. Moreover, this concentration also increased the blastocyst formation rate of vitrified porcine zygotes following SCNT. In addition, we reported that Ax treatment had no effect on total cell number per blastocyst. Unlike some other studies, supplementation of antioxidants during IVC can increase the blastocyst cell number. To further understand the beneficial effects of Ax supplementation on embryonic development, we detected the changes in gene expression for resultant blastocysts. It has been confirmed that vitrification can lead to abnormal gene expression in embryos; however, the expression patterns for the same gene may be different across various studies. In this study, the results of gene expression (*PCNA, POU5F1, CDX2, CPT1*, and *DMNT3b*) were similar to our previous study using the cloned embryos under the same experimental condition (6), and only *CDX2* gene expression was regulated by Ax treatment. *CDX2* plays a pivotal role in trophectoderm formation and has been reported to be highly expressed in vitrified mouse blastocysts (35). On the other hand, several endogenous antioxidant enzymes work to control oxidative stress and support cellular defense (36). We observed that vitrification of porcine zygotes did not affect the mRNA expression of *SOD1, CAT, GPX4*, and *SIRT1* in obtained blastocysts, with increased *SOD2* gene expression. In addition, these vitrified porcine zygotes cultured with Ax showed an increased mRNA expression of both *SOD2* and *GPX4* genes. Overall, these results suggested that Ax as a modified composition was indeed able to optimize the culture environment for embryonic development.

It is well known that ROS maintained within homeostatic range are involved in regulating cell growth and survival as central signaling molecules (37). However, excess ROS production can disturb the oxidant/antioxidant balance and then impairs various intracellular functions and structures in preimplantation embryos such as damaging proteins, lipids, DNA and organelles (38). In addition, the reduced GSH is the main component of intracellular antioxidant system and is an important biomarker for early embryonic development (39). Many evidences have suggested that vitrification triggers oxidant stress by increasing ROS production or decreasing GSH in embryos. In the present study, we also found similar results in the 4-cell embryos derived from vitrified porcine zygotes, but no significant change in both ROS and GSH levels for resultant blastocysts. The reasons for these results could be that the vitrified zygotes reached intracellular redox homeostasis during their subsequent development. On the other hand, inhibition of oxidant stress is one of the critical factors in the success of embryo culture and also contribute to improve the quality of vitrified embryos. The Ax with very powerful antioxidant properties has been proved to scavenge ROS in various cell types (40). For the vitrified porcine zygotes, Ax supplementation also effectively prevented ROS accumulation and promoted GSH content in obtained 4-cells, suggesting the improvement of embryo quality (41).

Mitochondria are the essential organelles involved in many cellular functions and have a pivotal role in oocyte competence and embryo development (42). Vitrification of oocytes or embryos has been reported to cause mitochondrial dysfunction such as influencing distribution, mitochondrial ROS production, membrane potential, ATP content and others (43, 44). For the vitrified porcine zygotes, although mitochondrial activity showed a decrease in 4-cell embryos, it was completely restored at the blastocyst stage. Moreover, Ax may be a potential target in mitochondria and enhances mitochondrial efficiency (45). In this study, Ax supplementation significantly increased the mitochondrial activity in both resultant 4-cells and blastocysts, indicating that Ax could activate mitochondrial function to improve the development and quality of embryos. Also the vitrified porcine zygotes cultured with Ax could retain ATP content in resultant blastocysts.

Lysosomal function plays a critical role in proteins hydrolysis and signal transduction, and is also important for oocyte meiosis and preimplantation embryonic development (46, 47). Moreover, cathepsin B as a member of the lysosomal protease family is directly connected with the quality of oocytes and embryos (48). A recent study has found that vitrification of mouse oocytes disrupts lysosomal function and stimulates cathepsin B activity (49). In the current study, we observed that vitrification of porcine zygotes could lead to an increase in lysosomal and cathepsin B activities at the 4-cell stage. Furthermore, lysosomal activity was restored at the blastocyst stage; however, cathepsin B activity failed to recover normal status. It has been reported in recent studies that Ax has inhibitory effects on cathepsin B activity in oocyte growth or maturation *in vitro* (22, 28). Similarly, this study also found that Ax supplementation was able to reduce the cathepsin B activity in both 4-cell embryos and blastocysts derived from vitrified porcine zygotes, but they still did not reach a level of the fresh control. In addition, the resultant blastocysts exhibited a lower lysosomal activity following Ax treatment during IVC. These results suggested that Ax supplementation might be beneficial in the recovery of abnormal lysosomal function induced by vitrification of embryos.

## Conclusion

In conclusion, our study demonstrated that Ax (1.5 μM) supplementation in the IVC medium exhibited potentially beneficial effects on the preimplantation embryonic development and embryo quality derived from vitrified porcine zygotes, at least partly, through modifying gene expression, alleviating oxidative stress, and ameliorating mitochondrial and lysosomal function. Therefore, Ax as a powerful exogenous antioxidant can be applied to establish more efficient IVC system for recovery culture of the vitrified porcine embryos or other reproductive biology field.

## Data Availability Statement

The original contributions presented in the study are included in the article/Supplementary Material, further inquiries can be directed to the corresponding author/s.

## Author Contributions

DX, BJ, and GW conceived the experiments. DX, BJ, BZ, JL, QH, HW, and GW conducted the experiments. DX, BJ, and GW performed statistical analysis, figure generation, and wrote the manuscript. GW reviewed the manuscript. All authors have read and agreed to the published version of the manuscript.

## Funding

This work was supported by Yunnan Applied Basic Research Projects (Nos. 202001AS070001 and 202101AT070213), Yunnan Young Academic Leader Program (No. 202005AC160004), and National Natural Science Foundation of China (No. 32160793).

## Conflict of Interest

The authors declare that the research was conducted in the absence of any commercial or financial relationships that could be construed as a potential conflict of interest.

## Publisher's Note

All claims expressed in this article are solely those of the authors and do not necessarily represent those of their affiliated organizations, or those of the publisher, the editors and the reviewers. Any product that may be evaluated in this article, or claim that may be made by its manufacturer, is not guaranteed or endorsed by the publisher.

## References

[1] Robles V Valcarce DG Riesco MF. The use of antifreeze proteins in the cryopreservation of gametes and embryos. Biomolecules. (2019) 9:181. 10.3390/biom9050181 31075977 PMC6571776

[2] Mandawala AA Harvey SC Roy TK Fowler KE. Cryopreservation of animal oocytes and embryos: current progress and future prospects. Theriogenology. (2016) 86:1637–44. 10.1016/j.theriogenology.2016.07.018 27555525

[3] Jin B Higashiyama R Nakata Y Yonezawa J Xu S Miyake M. Rapid movement of water and cryoprotectants in pig expanded blastocysts via channel processes: its relevance to their higher tolerance to cryopreservation. Biol Reprod. (2013) 89:87. 10.1095/biolreprod.112.107250 23966318

[4] Saragusty J Arav A. Current progress in oocyte and embryo cryopreservation by slow freezing and vitrification. Reproduction. (2011) 141:1–19. 10.1530/REP-10-0236 20974741

[5] Wu GQ Quan GB Shao QY Lv CR Jiang YT Zhao ZY. Cryotop vitrification of porcine parthenogenetic embryos at the early developmental stages. Theriogenology. (2016) 85:434–40. 10.1016/j.theriogenology.2015.09.015 26462660

[6] Jia BY Xiang DC Guo JX Jiao DL Quan GB Hong QH. Successful vitrification of early-stage porcine cloned embryos. Cryobiology. (2020) 97:53–9. 10.1016/j.cryobiol.2020.10.009 33065107

[7] Kopeika J Thornhill A Khalaf Y. The effect of cryopreservation on the genome of gametes and embryos: principles of cryobiology and critical appraisal of the evidence. Hum Reprod Update. (2014) 21:209–27. 10.1093/humupd/dmu063 25519143

[8] Bontekoe S Mantikou E van Wely M Seshadri S Repping S Mastenbroek S. Low oxygen concentrations for embryo culture in assisted reproductive technologies. Cochrane Database Syst Rev. (2012) 11:CD008950. 10.1002/14651858.CD008950.pub2 22786519 PMC11683526

[9] Soto-Heras S Paramio M. Impact of oxidative stress on oocyte competence for *in vitro* embryo production programs. Res Vet Sci. (2020) 132:342–50. 10.1016/j.rvsc.2020.07.013 32738731

[10] Zhang Z Mu Y Ding D Zou W Li X Chen B. Melatonin improves the effect of cryopreservation on human oocytes by suppressing oxidative stress and maintaining the permeability of the oolemma. J Pineal Res. (2021) 70:e12707. 10.1111/jpi.12707 33274466

[11] Gao L Du M Zhuan Q Luo Y Li J Hou Y. Melatonin rescues the aneuploidy in mice vitrified oocytes by regulating mitochondrial heat product. Cryobiology. (2019) 89:68–75. 10.1016/j.cryobiol.2019.05.005 31082378

[12] Ito J Shirasuna K Kuwayama T Iwata H. Resveratrol treatment increases mitochondrial biogenesis and improves viability of porcine germinal-vesicle stage vitrified-warmed oocytes. Cryobiology. (2020) 93:37–43. 10.1016/j.cryobiol.2020.02.014 32171796

[13] Chen H Zhang L Wang Z Chang H Xie X Fu L. Resveratrol improved the developmental potential of oocytes after vitrification by modifying the epigenetics. Mol Reprod Dev. (2019) 86:862–70. 10.1002/mrd.23161 31066155

[14] García-Martínez T Vendrell-Flotats M Martínez-Rodero I Ordóñez-León EA Álvarez-Rodríguez M López-Béjar M. Glutathione ethyl ester protects *in vitro*-maturing bovine oocytes against oxidative stress induced by subsequent vitrification/warming. Int J Mol Sci. (2020) 21:7547. 10.3390/ijms21207547 33066129 PMC7588878

[15] Moawad AR Tan SL Taketo T. Beneficial effects of glutathione supplementation during vitrification of mouse oocytes at the germinal vesicle stage on their preimplantation development following maturation and fertilization *in vitro*. Cryobiology. (2017) 76:98–103. 10.1016/j.cryobiol.2017.04.002 28412286

[16] Bahbah EI Ghozy S Attia MS Negida A Emran TB Mitra S. Molecular mechanisms of astaxanthin as a potential neurotherapeutic agent. Mar Drugs. (2021) 19:201. 10.3390/md19040201 33916730 PMC8065559

[17] Naguib YM. Antioxidant activities of astaxanthin and related carotenoids. J Agric Food Chem. (2000) 48:1150–4. 10.1021/jf991106k 10775364

[18] Ursoniu S Sahebkar A Serban MC Banach M. Lipid profile and glucose changes after supplementation with astaxanthin: a systematic review and meta-analysis of randomized controlled trials. Arch Med Sci. (2015) 11:253–66. 10.5114/aoms.2015.50960 25995739 PMC4424245

[19] Inoue M Tanabe H Matsumoto A Takagi M Umegaki K Amagaya S. Astaxanthin functions differently as a selective peroxisome proliferator-activated receptor gamma modulator in adipocytes and macrophages. Biochem Pharmacol. (2012) 84:692–700. 10.1016/j.bcp.2012.05.021 22732454

[20] Ambati RR Phang SM Ravi S Aswathanarayana RG. Astaxanthin: sources, extraction, stability, biological activities and its commercial applications–a review. Mar Drugs. (2014) 12:128–52. 10.3390/md12010128 24402174 PMC3917265

[21] Li R Wu H Zhuo WW Mao QF Lan H Zhang Y. Astaxanthin normalizes epigenetic modifications of bovine somatic cell cloned embryos and decreases the generation of lipid peroxidation. Reprod Domest Anim. (2015) 50:793–9. 10.1111/rda.12589 26280670

[22] Abdel-Ghani MA Yanagawa Y Balboula AZ Sakaguchi K Kanno C Katagiri S. Astaxanthin improves the developmental competence of *in vitro*-grown oocytes and modifies the steroidogenesis of granulosa cells derived from bovine early antral follicles. Reprod Fertil Develop. (2019) 31:272. 10.1071/RD17527 30071922

[23] Jang HY Ji SJ Kim YH Lee HY Shin JS Cheong HT. Antioxidative effects of astaxanthin against nitric oxide-induced oxidative stress on cell viability and gene expression in bovine oviduct epithelial cell and the developmental competence of bovine IVM/IVF embryos. Reprod Domest Anim. (2010) 45:967–74. 10.1002/9780813810898 19930137

[24] Namekawa T Ikeda S Sugimoto M Kume S. Effects of astaxanthin-containing oil on development and stress-related gene expression of bovine embryos exposed to heat stress. Reprod Domest Anim. (2010) 45:e387–91. 10.1111/j.1439-0531.2010.01584.x 20210879

[25] Kuroki T Ikeda S Okada T Maoka T Kitamura A Sugimoto M. Astaxanthin ameliorates heat stress-induced impairment of blastocyst development *in vitro*: -astaxanthin colocalization with and action on mitochondria-. J Assist Reprod Genet. (2013) 30:623–31. 10.1007/s10815-013-9987-z 23536152 PMC3663973

[26] Do LT Luu VV Morita Y Taniguchi M Nii M Peter AT. Astaxanthin present in the maturation medium reduces negative effects of heat shock on the developmental competence of porcine oocytes. Reprod Biol. (2015) 15:86–93. 10.1016/j.repbio.2015.01.002 26051456

[27] Jia BY Xiang DC Shao QY Zhang B Liu SN Hong QH. Inhibitory effects of astaxanthin on postovulatory porcine oocyte aging in vitro. Sci Rep. (2020) 10:20217. 10.1038/s41598-020-77359-6 33214659 PMC7677382

[28] Xiang DC Jia BY Fu XW Guo JX Hong QH Quan GB. Role of astaxanthin as an efficient antioxidant on the *in vitro* maturation and vitrification of porcine oocytes. Theriogenology. (2021) 167:13–23. 10.1016/j.theriogenology.2021.03.006 33743504

[29] Funahashi H Cantley TC Day BN. Synchronization of meiosis in porcine oocytes by exposure to dibutyryl cyclic adenosine monophosphate improves developmental competence following *in vitro* fertilization. Biol Reprod. (1997) 57:49–53. 10.1095/biolreprod57.1.49 9209079

[30] Jia BY Xiang DC Zhang B Quan GB Shao QY Hong QH. Quality of vitrified porcine immature oocytes is improved by coculture with fresh oocytes during *in vitro* maturation. Mol Reprod Dev. (2019) 86:1615–27. 10.1002/mrd.23249 31368632

[31] Yoshioka K Suzuki C Tanaka A Anas IM Iwamura S. Birth of piglets derived from porcine zygotes cultured in a chemically defined medium. Biol Reprod. (2002) 66:112–9. 10.1095/biolreprod66.1.112 11751272

[32] Hayashi T Kansaku K Abe T Ueda S Iwata H. Effects of resveratrol treatment on mitochondria and subsequent embryonic development of bovine blastocysts cryopreserved by slow freezing. Anim Sci J. (2019) 90:849–56. 10.1111/asj.13219 31067600

[33] Hara T Kin A Aoki S Nakamura S Shirasuna K Kuwayama T. Resveratrol enhances the clearance of mitochondrial damage by vitrification and improves the development of vitrified-warmed bovine embryos. PLoS ONE. (2018) 13:e204571. 10.1371/journal.pone.0204571 30335749 PMC6193637

[34] Pero ME Zullo G Esposito L Iannuzzi A Lombardi P De Canditiis C. Inhibition of apoptosis by caspase inhibitor Z-VAD-FMK improves cryotolerance of *in vitro* derived bovine embryos. Theriogenology. (2018) 108:127–35. 10.1016/j.theriogenology.2017.11.031 29207293

[35] Kazemi P Dashtizad M Shamsara M Mahdavinezhad F Hashemi E Fayazi S. Effect of blastocoel fluid reduction before vitrification on gene expression in mouse blastocysts. Mol Reprod Dev. (2016) 83:735–42. 10.1002/mrd.22681 27409768

[36] Mazur-Bialy AI Pocheć E. The time-course of antioxidant irisin activity: role of the Nrf2/HO-1/HMGB1 Axis. Antioxidants. (2021) 10:88. 10.3390/antiox10010088 33440644 PMC7827448

[37] Sies H Jones DP. Reactive oxygen species (ROS) as pleiotropic physiological signalling agents. Nat Rev Mol Cell Biol. (2020) 21:363–83. 10.1038/s41580-020-0230-3 32231263

[38] Guerin P El MS Menezo Y. Oxidative stress and protection against reactive oxygen species in the pre-implantation embryo and its surroundings. Hum Reprod Update. (2001) 7:175–89. 10.1093/humupd/7.2.175 11284661

[39] Hansen JM Harris C. Glutathione during embryonic development. Biochim Biophys Acta. (2015) 1850:1527–42. 10.1016/j.bbagen.2014.12.001 25526700

[40] Afzali A Amidi F Koruji M Nazari H Gilani MAS Sanjbad AS. Astaxanthin relieves busulfan-induced oxidative apoptosis in cultured human spermatogonial stem cells by activating the Nrf-2/HO-1 pathway. Reprod Sci. (2021) 29:374–94. 10.1007/s43032-021-00651-x 34129218

[41] Yu W Chen C Peng Y Li Z Gao Y Liang S. Schisanhenol improves early porcine embryo development by regulating the phosphorylation level of MAPK. Theriogenology. (2021) 175:34–43. 10.1016/j.theriogenology.2021.08.019 34481228

[42] May-Panloup P Boguenet M El Hachem H Bouet P Reynier P. Embryo and its mitochondria. Antioxidants. (2021) 10:139. 10.3390/antiox10020139 33498182 PMC7908991

[43] Gualtieri R Kalthur G Barbato V Di Nardo M Adiga SK Talevi R. Mitochondrial dysfunction and oxidative stress caused by cryopreservation in reproductive cells. Antioxidants. (2021) 10:337. 10.3390/antiox10030337 33668300 PMC7996228

[44] Iwata H. Resveratrol enhanced mitochondrial recovery from cryopreservation-induced damages in oocytes and embryos. Reprod Med Biol. (2021) 20:419–26. 10.1002/rmb2.12401 34646069 PMC8499604

[45] Krestinina O Baburina Y Krestinin R. Mitochondrion as a target of astaxanthin therapy in heart failure. Int J Mol Sci. (2021) 22:7964. 10.3390/ijms22157964 34360729 PMC8347622

[46] Tsukamoto S Tatsumi T. Degradation of maternal factors during preimplantation embryonic development. J Reprod Dev. (2018) 64:217–22. 10.1262/jrd.2018-039 29695651 PMC6021607

[47] Wang Y Xu Y Ju J Liu J Sun S. Fumonisin B1 exposure deteriorates oocyte quality by inducing organelle dysfunction and DNA damage in mice. Ecotox Environ Saf. (2021) 223:112598. 10.1016/j.ecoenv.2021.112598 34388657

[48] Li J Maeji M Balboula AZ Aboelenain M Fujii T Moriyasu S. Dynamic status of lysosomal cathepsin in bovine oocytes and preimplantation embryos. J Reprod Develop. (2020) 66:9–17. 10.1262/jrd.2019-115 31685761 PMC7040204

[49] Balboula AZ Schindler K Kotani T Kawahara M Takahashi M. Vitrification-induced activation of lysosomal cathepsin B perturbs spindle assembly checkpoint function in mouse oocytes. Mol Hum Reprod. (2020) 26:689–701. 10.1093/molehr/gaaa051 32634244 PMC7828578

