# Supplementary material for: Astaxanthin Supplementation Improves the Subsequent Developmental Competence of Vitrified Porcine Zygotes
Source: Front Vet Sci. 2022 Apr 1;9:871289. doi: 10.3389/fvets.2022.871289 (PMC9011099; doi:10.3389/fvets.2022.871289)
Supplement: Supplementary file 1 [file Table_1.DOCX]

**Supplementary Table S1 |** Primers used for gene expression analysis.

| **Gene symbol** | **Primer sequences (5′–3′)** | **Product size (bp)** | **GenBank number** |
| --- | --- | --- | --- |
| *GAPDH* | F: GACCCCTTCATTGACCTCCA | 131 | NM_001206359.1 |
|  | R: TGGAAGATGGTGATGGCCTT |  |  |
| *PCNA* | F: AGAGGAGGAAGCAGTTACCAT | 104 | NM_001291925.1 |
|  | R: CTGTAGGAGAGAGTGGAGTGG |  |  |
| *POU5F1* | F: GCGGACAAGTATCGAGAACC | 200 | XM_021097869.1 |
|  | R: CCTCAAAATCCTCTCGTTGC |  |  |
| *CDX2* | F: TCCTTTAGTGCTGGCAGGAA | 108 | NM_001278769.1 |
|  | R: CAACCAGTCGATGCATCCTG |  |  |
| *CPT1* | F: GGACCGCCACCTGTTCTG | 172 | AF288789 |
|  | R: CCCTCCGCTCGACACATAC |  |  |
| *DNMT3b* | F: TTACCAAAACCACAGTGCCG | 137 | NM_001348900.1 |
|  | R: TTACCAAAACCACAGTGCCG |  |  |
| *SOD1* | F: TCCATGTCCATCAGTTTGGA | 131 | NM_001190422.1 |
|  | R: AGTCACATTGCCCAGGTCTC |  |  |
| *SOD2* | F: AAGCCATCAAACGCGACTTT | 107 | NM_214127.2 |
|  | R: CCTTGTTGAAACCGAGCCAA |  |  |
| *CAT* | F: ACATGGTCTGGGACTTCTGG | 100 | NM_214301 |
|  | R: TCATGTGCCTGTGTCCATCT |  |  |
| *GPX4* | F: ATTCTCAGCCAAGGACATCG | 93 | NM_214407.1 |
|  | R: CCTCATTGAGAGGCCACATT |  |  |
| *SIRT1* | F: TCACTGTGGTAGAGCTTGCA | 112 | NM_001145750 |
|  | R: ACGCTCCCCAAGTTGAAGTA |  |  |
